# Spatio-Temporal Variation in Length-Weight Relationships and Condition of the Ribbonfish *Trichiurus lepturus* (Linnaeus, 1758): Implications for Fisheries Management

**DOI:** 10.1371/journal.pone.0161989

**Published:** 2016-08-31

**Authors:** Abdullah AL Nahdi, Carlos Garcia de Leaniz, Andrew J. King

**Affiliations:** 1 Department of Biosciences, College of Science, Swansea University, Singleton Park, Swansea, United Kingdom; 2 Marine Science and Fisheries Centre, Fisheries Research Directorate, Ministry of Agriculture and Fisheries Wealth, Muscat, Sultanate of Oman; Department of Agriculture and Water Resources, AUSTRALIA

## Abstract

Knowledge of length-weight relationships for commercially exploited fish is an important tool for assessing and managing of fish stocks. However, analyses of length-weight relationship fisheries data typically do not consider the inherent differences in length-weight relationships for fish caught from different habitats, seasons, or years, and this can affect the utility of these data for developing condition indices or calculating fisheries biomass. Here, we investigated length-weight relationships for ribbonfish *Trichiurus lepturus* in the waters of the Arabian Sea off Oman collected during three periods (2001–02, 2007–08, and 2014–15) and showed that a multivariate modelling approach that considers the areas and seasons in which ribbonfish were caught improved estimation of length-weight relationships. We used the outputs of these models to explore spatio-temporal variations in condition indices and relative weights among ribbonfish, revealing fish of 85–125 cm were in the best overall condition. We also found that condition differed according to where and when fish were caught, with condition lowest during spring and pre-south-west monsoon periods and highest during and after the south-west monsoons. We interpret these differences to be a consequence of variability in temperature and food availability. Based on our findings, we suggest fishing during seasons that have the lowest impact on fish condition and which are commercially most viable; such fishery management would enhance fisheries conservation and economic revenue in the region.

## Introduction

Length-weight (LW) relationships are commonly used in fisheries science to derive a quantitative measure of biomass [1]. The relative relationship between fish body length and weight is used as a proxy for fish condition, based on the assumption that heavier fish of a given length are in better condition [2]. As such, estimation of LW relationships can provide important information to fisheries managers and is helpful in understanding both growth rates of fish populations and their dynamics [3]. The relationship between fish length and weight is also important for determining or predicting the condition or relative “wellness” of fish communities [4–6].

The arithmetical form of the relationship between length (L; in cm) and weight (W; in g) can be described by the power function (*W* = *aL*^*b*^), and the parameters *a* and *b* can be estimated from linear regression applied to the log-transformed variables (log *W* = log *a* + *b* log *L*). Generally, LW relationships are modelled assuming error structures on the observed weights are log-normally distributed, but this approach can result in biased estimates for stock assessment calculations [7]. As an alternative, it is possible to consider and control for the potential non-independence of estimated LW relationships for fish within and between different ‘groups’ by employing a mixed model approach [8].

This study focused on a commercially important marine species, the ribbonfish (*Trichiurus lepturus*). The ribbonfish is a benthopelagic species found from continental shelf to inshore waters of approximately 350 m in depth; it moves in dense shoals, and feeds on several species of small fishes, squids and crustaceans [9]. Adults feed on plankton near the sea surface at night and return back to the bottom zone during the day [10]. Owing to their coastal distribution, they are often heavily targeted in artisanal and commercial fisheries [11].

We investigated LW relationships and condition of ribbonfish in waters of the Arabian Sea off Oman. National annual reports show an average of 6181 tonnes have been caught annually since 1995, and in 2011–2012 ribbonfish was the most landed species [12]. In our analyses, we account for spatio-temporal variations in LW relationships, which to our knowledge is the first study to do this at a regional scale. First, we compared the accuracy of different models to explain variation in length-weight data and then compared condition factor indices and relative weights among fish caught in different locations and during different seasons. We expected LW relationships to vary with location since fish growth is typically influenced by local resource availability [13]. We also expected LW relationships to vary within seasons since the Arabian Sea is annually affected by two seasonal monsoons, generated from upwelling by southwest winds and downwelling by northwest winds, resulting in changes in productivity along the Omani coast [14]. We then use this information to provide recommendations for sustainable management of this important fishery in the region.

## Material and Methods

### Ethics statement

Our study used (dead) fish collected by Marine Science and Fisheries Centre (MSFC) researchers from landing sites and did not involve any protected or endangered species. Permission and approval to collect and use samples were given by the MSFC, who are responsible for monitoring commercial fisheries and provide advice to the Ministry of Agriculture and Fisheries Wealth in Oman. No further authorisations or approvals were required for this research.

### Sampling

**Fig 1:**
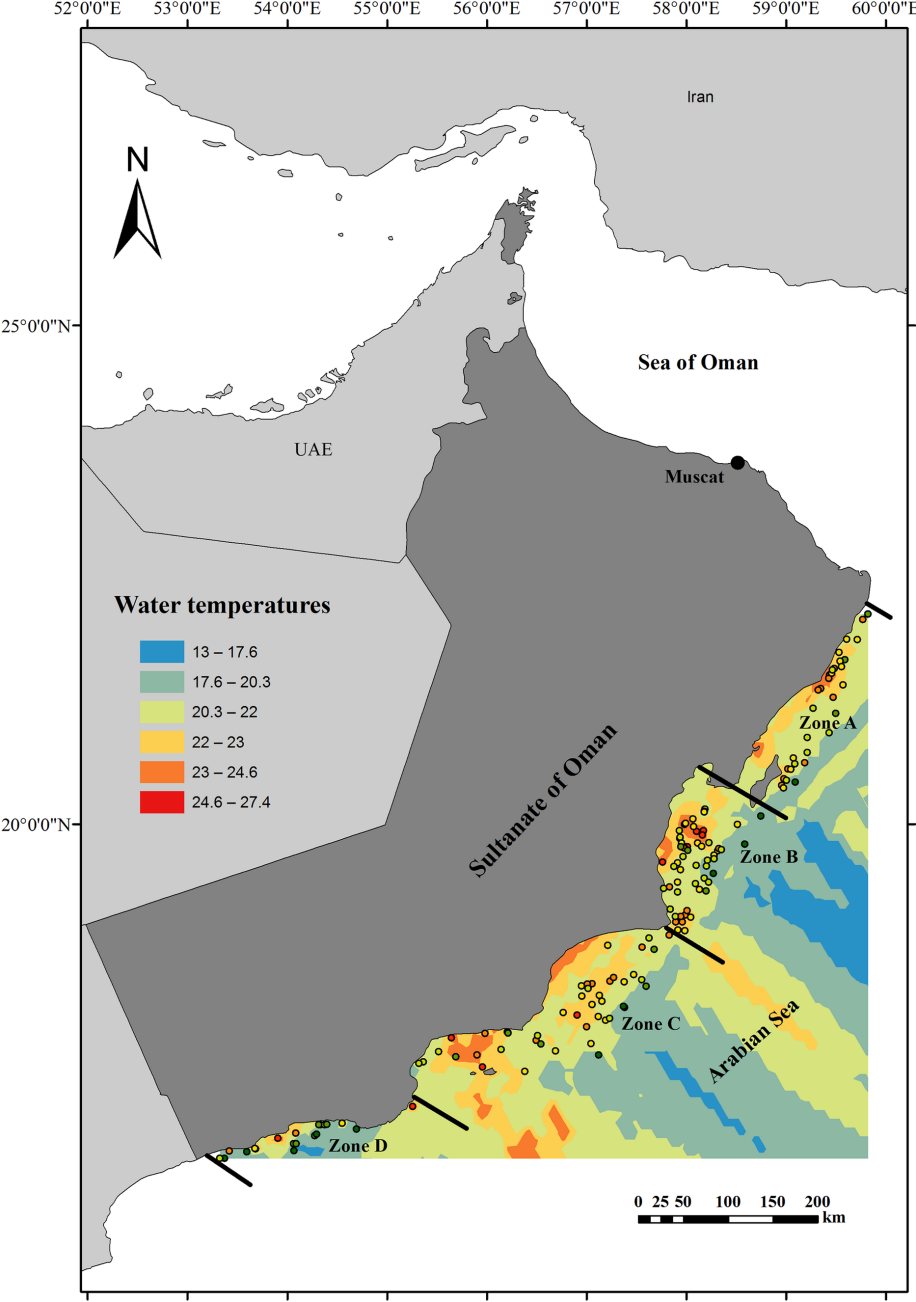
**Study area:** Study zones (A-D) are separated by the thick line. Bottom water temperature profiles are shown by a colour scale based upon mean average seasonal temperatures at sampling collection stations (black dots) used by the Arabian Sea commercial species survey. Background digital map is open-source [17].

Length-weight measurements of a total 2557 ribbonfish sampled from the Arabian Sea off the Omani coast were taken from historical records collected during 2001–2002 (period I), 2007–2008 (period II) and by the authors during 2014–2015 (period III). During periods I and III, fresh samples were collected opportunistically from major landing sites along the Arabian Sea coast by scientists at MSFC in Oman. Period II fish were caught by a MSFC research vessel during an stratified survey carried out between 21°50' and 16°45'N longitude of the Arabian Sea off Oman, from Ras Al Hadd to the south of Oman in Salalah (Fig 1). In all cases, fish were processed fresh and sexed. Total length (TL) was measured to the nearest 0.1 cm and total weight (Wt) was measured to the nearest 1 g. Maturity stages were recorded [15] for 840 randomly selected fish collected during period I and period III.

### Spatio-temporal data

Sampling periods covered the major seasons and monsoon periods in the region, namely ‘pre-southwest monsoon’ (pre-SW), ‘post-southwest monsoon’ (post-SW), ‘northeast monsoon’ (NE) and ‘spring monsoons’ (Spring), and the region was divided up into four major zones (A–D) that followed the Omani coastline (Fig 1). The sea temperature in the region ranged from 16.8°C to 27.4°C and mean ± SD and temperature (bottom water) profiles for the region were generated using a semivariogram model [16] based upon recordings (±0.1°C) at local sampling stations (Fig 1). The warmest average temperatures were recorded during the autumn inter-monsoon season (September to November). Waters are cooler over the southern area (zone D), and particularly so during pre-SW monsoon due to water layers mixing through upwelling. Temperatures are warmer towards the north, although a few pockets of cool water occurred during pre-SW monsoon (Fig 1).

### Length-weight relationships

All analyses were conducted using the R statistical software version 3.2.0 [18] and the Fisheries Stock Assessment package [19] to derive length-weight relationship parameters. First, we conducted a basic linear regression (model 1) in the form presented in Eq (1), where *Wt* is total weight, *TL* is total length, *α* is the regression intercept, and *β* is the regression slope.

$${log}_{10}(Wt)={log}_{10}(\alpha )+{\beta log}_{10}(TL)$$ (1)

Because several factors may affect fish length and weight, this can introduce bias (error) to the estimates, and therefore, we extended the model (Eq 2) to include an error term, with error assumed to be independent and normally distributed:

$${log}_{10}(Wt)={log}_{10}(\alpha )+{\beta log}_{10}(TL)+\epsilon_{i}$$ (2)

where ε is the error associated with combining data for fish from different seasons (model 2) or zones (model 3) at the level of fish individuals population. To systematically control for differences in season and zones with respect to length, we then fitted models with interaction terms with fish length and season (model 4) or zone (model 5). This allowed us to model LW relationships, including factors separately or as interactions (model 6) to test if the relationship between length and weight (i.e. slopes) was statistically different across zones and seasons. In our final model (model 7), we used an ANCOVA interaction approach [8]. This allowed us to test the relationship between weight and length while controlling for other potentially confounding variables, and considering interactions among these. We fitted the following terms as fixed factors: log_10_TL (continuous); zones (A-D); season (pre-SW, post-SW, NE, and spring) including biologically relevant (such as fish size) two-way interactions among these terms. We did not include day of sampling as random effect in the models because the records of fish at the day level are not available for all periods. However, we included period (I, II, III) to control for any potential differences across the different sampling times, as well as sex (male, female) and fish maturity (immature, mature) which could also affect LW relationships.

The Akaike Information Criterion (AIC) was used to select among models, and also to select the most appropriate combination of fixed and random effects in model 7 [20]. The final, selected model was the model with the lowest AIC value, and we used estimates from this model to explore condition indices in the population.

### Condition indices

We estimated Fulton’s condition factor (*K*) [21] which assumes the shape of fish does not change with size (i.e. isometric growth) by the following equation:

$$K=\frac{Wt}{{TL}^{3}}*\;10^{3}$$ (3)

where *Wt* is total weight of fish and *TL* is the observed total length. The constant is a scaling factor. We evaluated the mean of *K* for each of our class intervals or length-groups.

Relative condition factor (*Kn*) [22] was also obtained. *Kn* allows for the offset of variations in fish condition with individual growth [23], by comparing the sampling weight of a given fish to the mean weight for given length class, using the equation:

$$Kn=\frac{Wt}{{TL}^{b}}$$ (4)

where *b* is the back transformed (i.e. anti-logged) [24] slope estimated from our best fitting model (see above).

For both *Kn* and *K* we then investigated differences between observed and predicted fish weights (logarithmic scale) and plotted these residuals against individual total length from our best fitting model (see above) for different zones and seasons. This allowed us to estimate those fish with ‘good’ (above 95) condition factors.

Finally, we employed the technique of Murphy [25] to calculate fish relative weight *Wr* [2, 4] as the ratio of the fish weight *Wt* observed to the standard weight *W*_*S*_ for each fish at class interval lengths [26], as:

$$Wr=\frac{Wt}{Ws}$$ (5)

*W*_*S*_ is constructed by series of steps, starting by predicting log_10_Wt on log_10_TL from each population (in this study zone population), and this linear regression was run separately for mature and immature fish since sex organs and maturity stage will impact on weight and thus estimates of condition [27]. We then re-transformed calculated values to weight and the 75^th^ percentile regression-line–percentile (RLP) was then estimated in each 5-cm length class intervals with no length-related biases [25]. The product was then log transformed and re-regressed with log_10_TL to estimate the critical parameter for the standard weight Ws. Hence, Wr was used to explore differences among zones and seasons by detecting the recommended Wr target range of 95–105 for balanced fish stocks [3]. Linear model analyses were conducted to detect the mean significant differences of Wr among studied populations and Tukey’s honestly significant difference (HSD) test was used for pair comparisons and to identify differences in Wr between zones and seasons.

## Results

### Length-weight relationships

**Fig 2:**
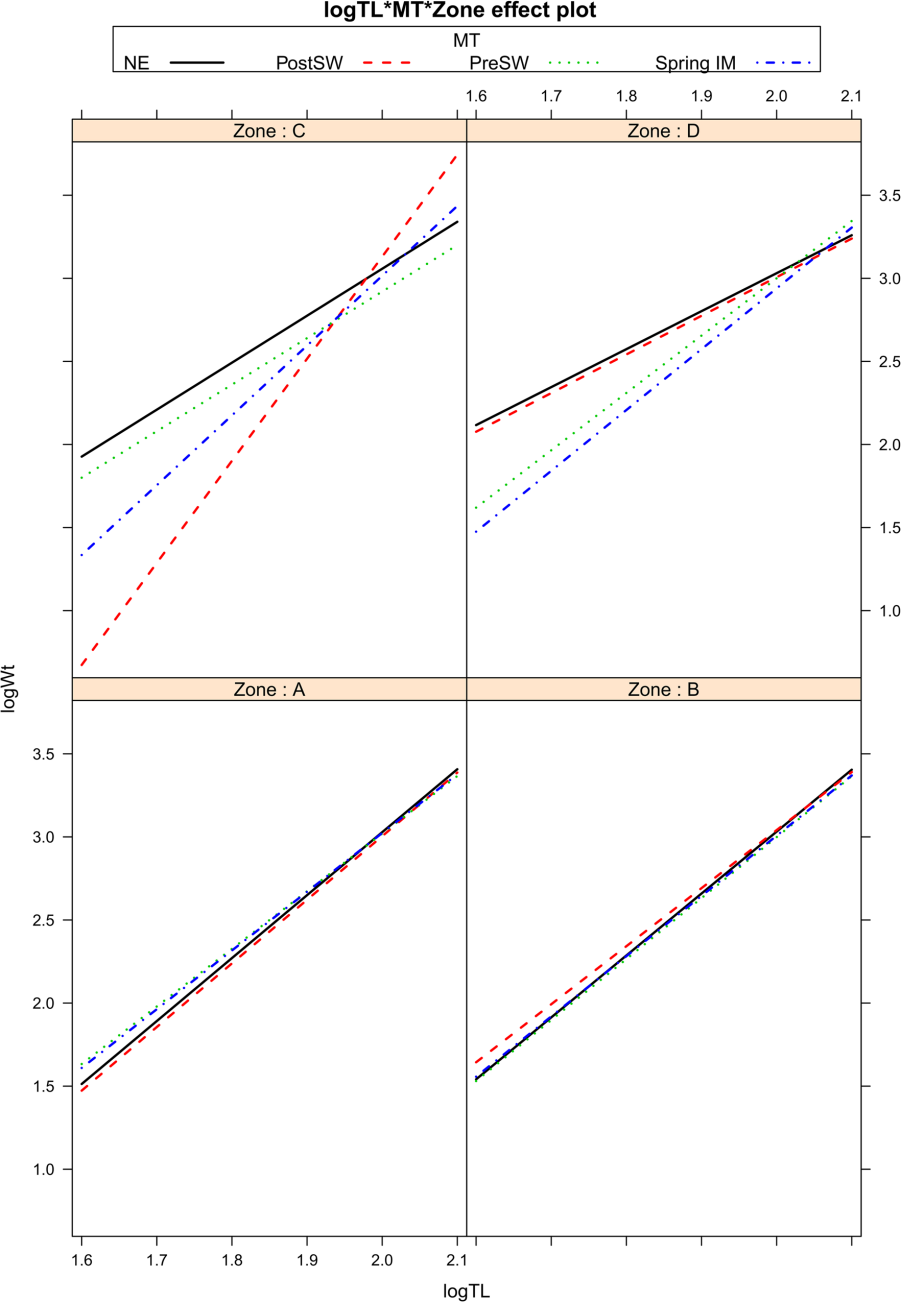
Length-weight relationships for ribbonfish in Oman according to seasons and zones. Effect function plot for linear model interactions of log10 transformed total weight (Wt) on log10 transformed total length (TL) data of *Trichiurus lepturus* in the Arabian Sea caught from four zones (A to D). The four seasons are: pre-SW: pre-southwest monsoon, post-SW: post-southwest monsoon, NE: northeast monsoon, Spring: spring monsoon.

Log_10_ transformed weight significantly predicted lengths in all models tested (Table 1). The model that provided the best fit (Table 2) considered an interaction between fish weight, location (zones) and timing (season), since LW relationships in zone C during the spring season were significantly different from other zones and/or seasons (Table 2; Fig 2). Controlling for the period in which data were collected (i.e. period I, II, III) and fish maturity also improved the model fit (Table 2), but as we found no significant interactions with sex (linear regression; estimated coefficient = 1.122, S.E. = 5.683, t-value = 0.197, p = 0.843), these were removed from the models. We therefore used estimates for *a* and *b* from our best model for further analyses, as follows:

$${log}_{10}(Wt)=-4.545+{3.788\;log}_{10}(TL)$$ (6)

**Table 1:** Comparison of models used to evaluate length weight relationship among four zones during four seasons for ribbonfish populations in Arabian Sea.

Model	Df	R^2^	SE	F	AIC
(1) length	3	0.940	0.0711	4.02	-6257.91
(2) length + season	6	0.941	0.0706	1.02	-6289.657
(3) length + zones	6	0.942	0.0701	1.04	-6328.526
(4) length + season*length	9	0.941	0.0703	5886	-6311.040
(5) length + zones*length	9	0.945	0.0685	6211	-6440.598
(6) length + zones*season	17	0.945	0.0686	2893	-6440.394
(7) full model with all terms	34	0.949	0.0632	1612	-6829.631

df: degrees of freedom, R^2^: R-square, SE: standard errors, F: F-statistic, and AIC: Akaike Information Criterion estimations.

**Table 2:** Output of best fitting model investigating length-weight relationships for ribbonfish *Trichuirus lepturus* in the Arabian Sea.

	Df	Coefficients	S.E.	t -value	P
**Length**	1	3.788	0.566	6.69	<0.001
**Maturity**	1	0.022	0.005	4.928	<0.001
**Period**	2				
Period I (reference)		0.000	0.000		
Period II		-0.693	0.004	-15.502	<0.001
Period III		-0.026	0.005	-4.928	<0.001
**Terms interaction with length**	34				
NE:ZoneA (Reference)		0.000	0.000		
PostSW:ZoneB		-0.051	0.586	-0.086	0.931
PreSw: ZoneB		0.323	0.599	0.540	0.589
Spring IM:ZoneB		0.448	0.576	0.778	0.437
PostSW:ZoneC		3.192	5.286	0.604	0.546
PreSw:ZoneC		0.005	0.781	0.007	0.995
Spring IM:ZoneC		1.634	0.738	2.213	0.027
PostSW:ZoneD*		NA	NA	NA	NA
PreSw:ZoneD		1.677	0.876	1.913	0.056
Spring IM:ZoneD		1.914	0.882	2.171	0.030

preSW: pre-southwest monsoon, postSW: post-southwest monsoon, NE: northeast monsoon, Spring: spring monsoon. Zones are detailed in Fig 1. Results from ANCOVA model based on length, maturity, and interactions with season and zone. Df: degrees of freedom, coefficients: parameter estimated, SE: standard errors, t-value: statistic t value, and P: p-value, p<0.001 indicates a significant effect.

*NA is insufficient data to estimate an effect.

### Condition indices

**Fig 3:**
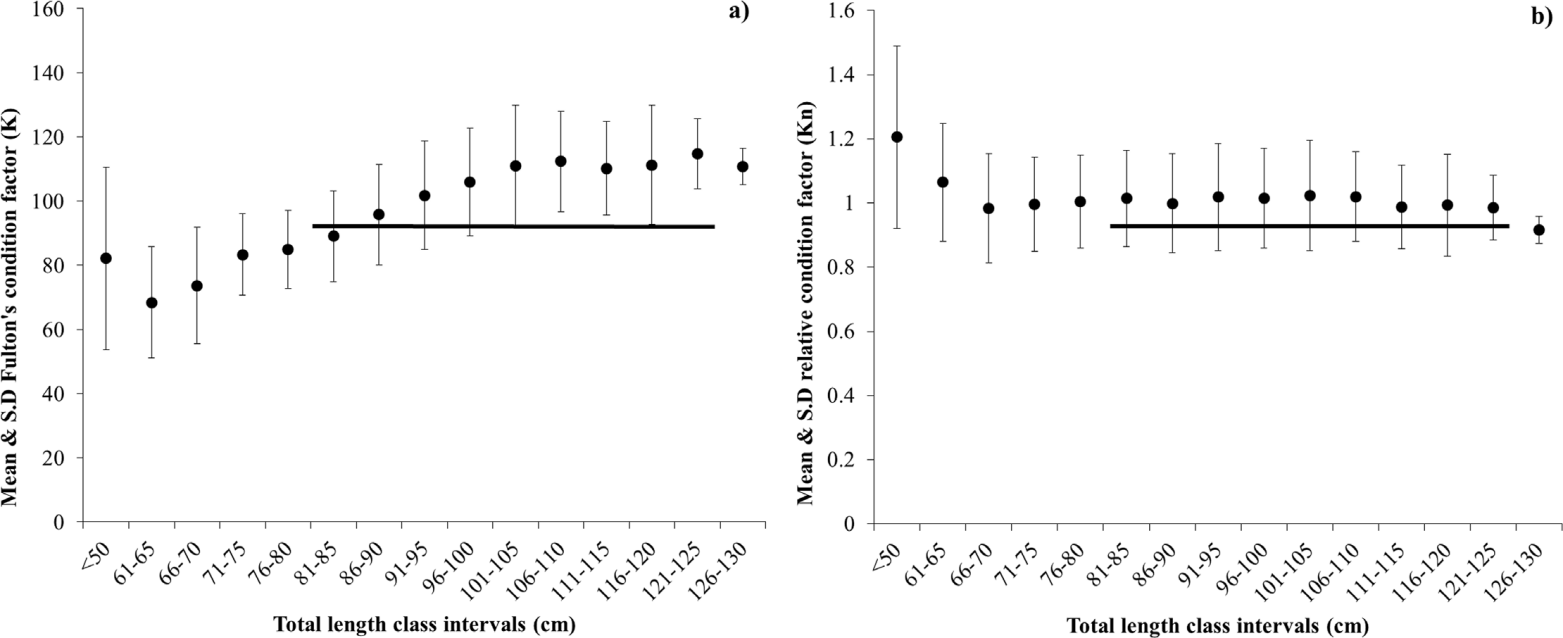
Condition factors for ribbonfish a) Fulton’s condition factor (K; Eq 3) and b) relative condition factor (Kn; Eq 4) means across total length class intervals of *Trichuirus lepturus* in the Arabian Sea. The horizontal line represents fish length ranges that have the highest Fulton’s and relative condition factors.

We used the best fitting model LW parameters to explore fish condition factors at total length class intervals. Fulton’s condition factor (K) ranged from 68.41 to 114.76 with a mean ± SD of 97 ±15.57 (Fig 3A). Relative condition factors (Kn) ranged between 0.87 to 1.25 and with mean ± standard deviation (SD) of 1.01 ±0.079 (Fig 3B).

Relative weight equations provided functionally similar results to our LW models:

$${log}_{10}(Ws)=-4.386+{3.712\;log}_{10}(TL)$$ (7)

**Fig 4:**
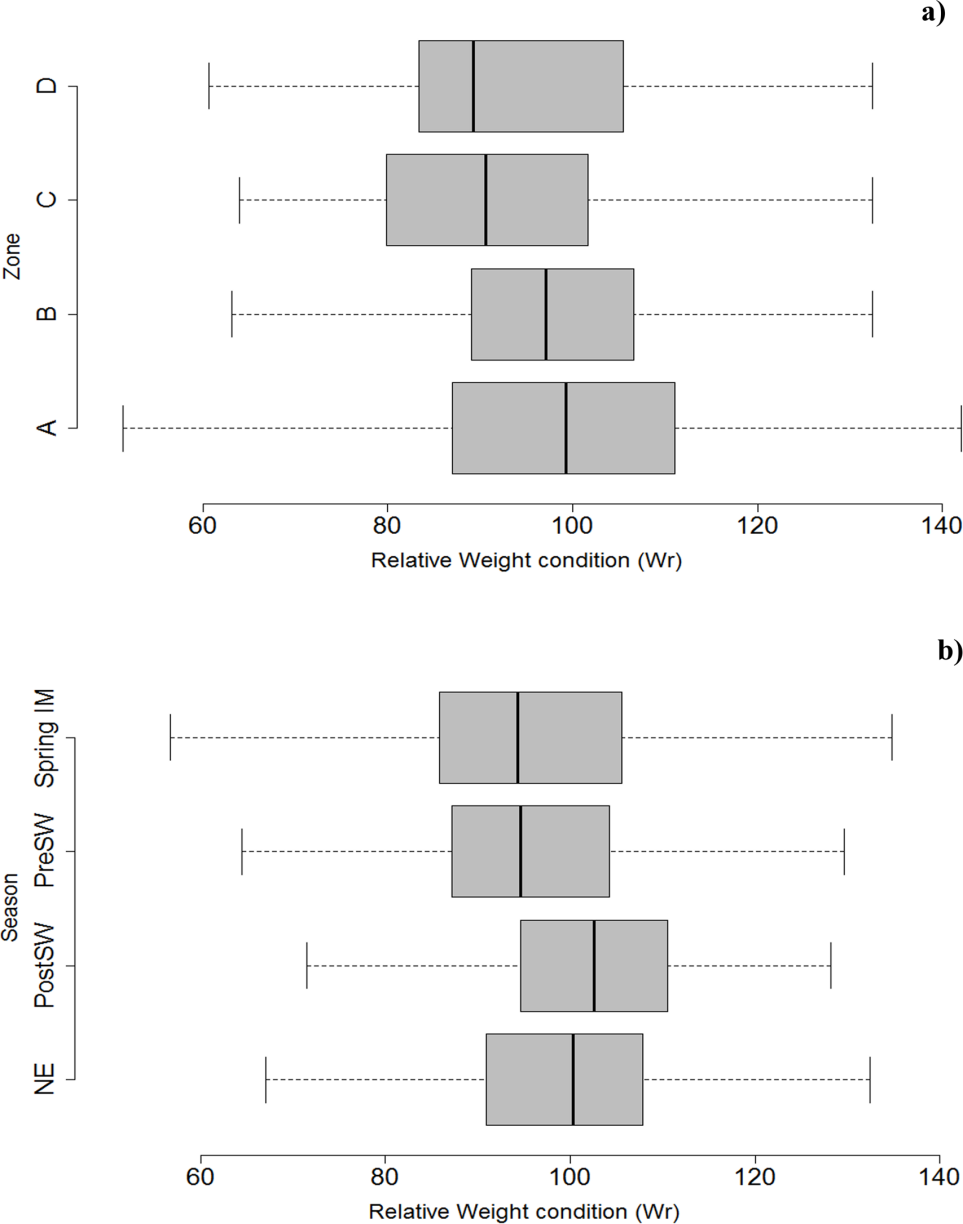
Relative weights of ribbonfish by zone and season. Box plot of relative weight condition factor (Wr; Eq 5) based on regression line of predicted standard weight (Ws) at 75th percentile, among zones (A to D) of ribbonfish *Trichuirus lepturus* populations in the Arabian Sea. The four seasons are: pre-SW: pre-southwest monsoon, post-SW: post-southwest monsoon, NE: northeast monsoon, Spring: spring monsoon.

As with LW models, we found significant differences in Wr between zones (Fig 4A; Table 3) and seasons (Fig 4B; Table 3).

**Table 3:** Tukey’ test (HSD) for pairwise comparisons for relative weight condition differences for ribbonfish *Trichuirus lepturus* caught in different regions of the Arabian Sea.

Pairwise comparisons	Coefficient	SE	t- value	P value (>|t|)
**Length (TL)**	0.116	0.0218	5.332	<0.001
**Zone**				
B–A	-0.018	0.0090	-2.020	0.163
C–A	-0.087	0.0109	-7.936	**<0.001**
D–A	-0.070	0.0218	-3.204	**0.006**
C–B	-0.069	0.0084	-8.189	**<0.001**
D–B	-0.052	0.0207	-2.507	0.051
D–C	-0.017	0.0216	0.781	0.851
**Monsoon**				
PostSW–NE	0.489	1.342	0.365	0.983
PreSW–NE	-4.837	1.168	-4.143	**<0.001**
Spring IM–NE	-5.486	0.945	-5.808	**<0.001**
PreSW–PostSW	-5.327	1.309	-4.070	**<0.001**
Spring–PostSW	-5.976	1.114	-5.363	**<0.001**
Spring IM–PreSW	-0.649	0.897	-0.723	0.884

Zones are detailed in (Fig 1) and four seasons; preSW: pre-southwest monsoon, postSW: post-southwest monsoon, NE: northeast monsoon, Spring: spring monsoon. Results from linear model tests, coefficients: pairwise subtraction parameter, SE: standard errors, t-value: statistic t value, and P value (>|t|): p-value, p<0.001 indicates a significant effect presented in bold fonts. Applying a Bonferroni adjustment to our P-value for multiple testing does not alter any of the reported significant differences.

**Fig 5:**
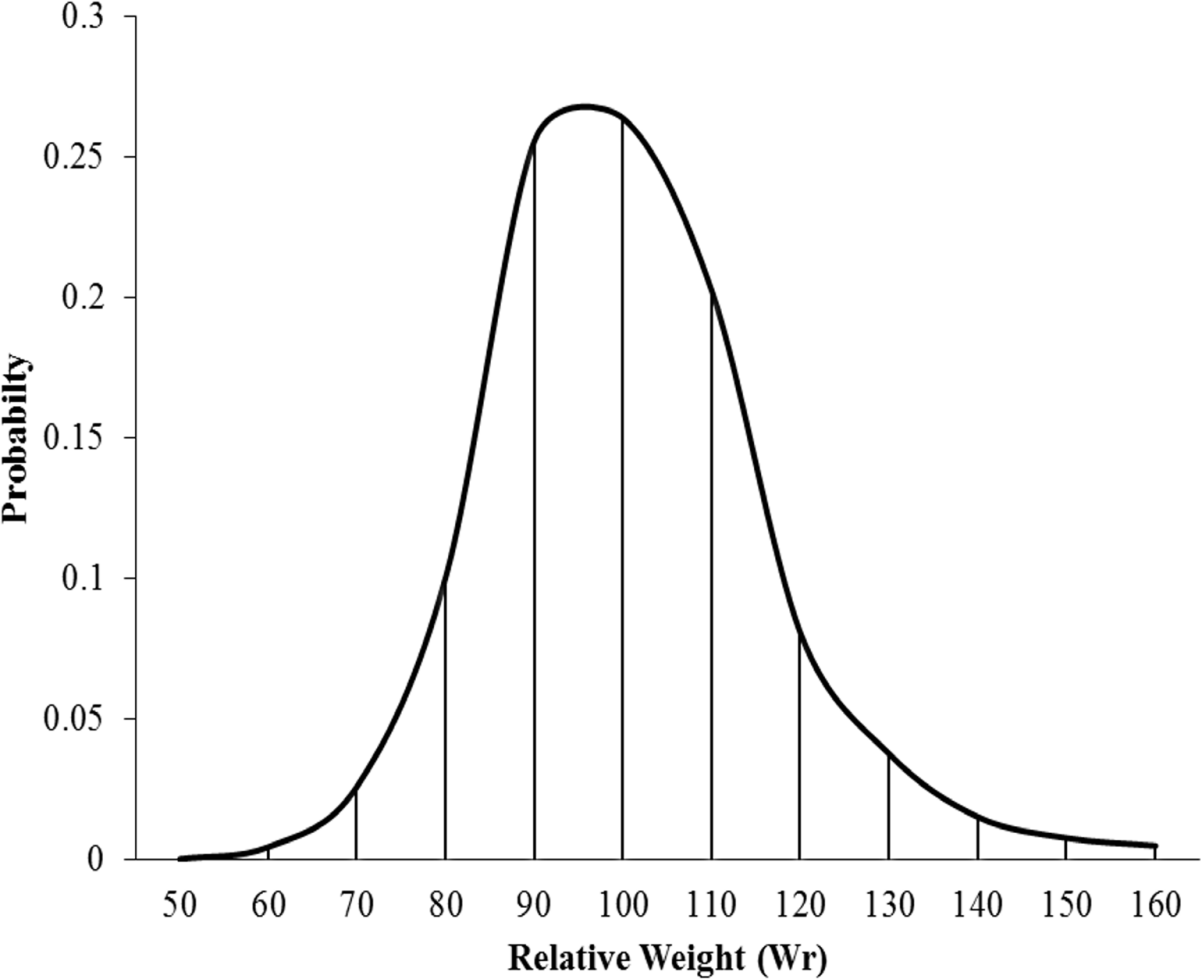
Frequency (probability) distribution of relative weight (Wr) means in combined populations.

Based on the distribution proportion of Wr values in our data, approximately 50% of Wr values fell within the range of 90–100, which is representative of a ‘healthier fish condition’ according to multiple sources [4, 25, 28]. Lower Wr condition represents approximately 13% of the sample, and very high condition above 100 represented more than 35% of samples (Fig 5).

## Discussion

We have shown that variation in LW relationships and condition of ribbonfish are best understood by considering and controlling for the potential non-independence of estimated LW relationships for fish within and between different groups simultaneously. Specifically, we found that the ANCOVA model which controlled for differences in length within and between fish classes produced the most parsimonious model output [8] to derive the parameters *a* and *b*. These parameters were outside of the limits reported by Froese [23], but this is perhaps not surprising given the atypical body shape of this species. We then used these estimates to model and predict differences in fish condition. We discuss each of our major findings in turn.

Our analyses revealed that the LW relationships varied significantly among seasons and zones for all fish sizes, suggesting there are strong spatial and temporal effects with years (Table 1), but little variation across years. Differences according to zones are likely to be caused by local differences in environmental factors such as temperature (Fig 1), habitat type, and/or potential inherent differences in fish behaviour and physiology across the study region [27]. Our categories for ‘season’ represent monsoon periods, which can drastically alter environmental conditions and thus likely impact fish condition. For example, Piontkovski and Al-Oufi [29] reported that the upper 30 m of water column increased by 1.2°C over the last five decades during the south-west monsoons in our study region. Such regional environmental changes may thus be driving variance in fish community condition [30].

We found that relative condition factor Kn and Fulton’s condition factor K varied significantly among length classes, with the best condition among fish from ~85 to 125 cm TL, representing mature adult individuals [31, 32]. Our relative weight analysis also revealed that fish in the northern area are heavier and larger compared to those caught in the southern zones. This is may be partly due to the south-west monsoon, which causes an upwelling in this area, potentially increasing prey available to ribbonfish. Seasonal changes appear to be critical to fish condition in our study; we found ribbonfish were in better condition from September to February and in poorest condition during the onset of SW monsoon. These seasons are associated with drastic changes in water temperatures [33–35]. For example, in our study region, coastal seawater temperatures rise before the onset of the south-west monsoon (25° to 28°C), before decreasing abruptly during the south-west monsoon (16° to 21°C) [35]. Such changes are typically synchronised with periods of high fish (prey) abundance [23] and thus indirectly affect ribbonfish condition which may account for the variation we observed in LW relationships. Several other commercial fish species are reported to show a similar body condition pattern during upwelling (monsoon) events, and have also been interpreted in relation to seasonal variation in food availability [36, 37].

Our results suggest that ribbonfish in the northern fisheries of the Arabian Sea display better condition during the post SW monsoon than individuals of the same size found further south. This suggests that it would be pertinent to open fisheries in the autumn season (post SW monsoon) in zones A and B with a catch slot size of 85–125 cm TL. This would act to relax fishing pressure on those fish that are important to recruitment and reduce the total number of spawners removed from the stock [38, 39]. This strategy would also exploit the better-conditioned individuals of the stock [40]. Overall, this should increase economic revenue via reduced exploitation of smaller, poorer conditioned fish, and increased commercial income from landing fish of greater weight and quality. For example, if we compare adult fish (e.g. 80 cm) caught in the post-SW monsoon in zone A (good condition) with fish caught in the Spring monsoon in zone D (poor condition), the former will result in a 37% better price per kg for the fisherman.

In conclusion, our multivariate modelling approach that considers the areas and seasons in which ribbonfish were caught improves estimation of LW relationships. We used the outputs of these models to explore spatio-temporal variations in condition indices and relative weights among ribbonfish TL, revealing fish of 85–125 cm were overall in the best condition. We also found that condition differed according to where and when fish were caught. This suggests that the condition of ribbonfish in the north-west of the Arabian Sea is affected by seasonal variation in food availability. Our study also indicates that condition indices, for example Fulton’s K condition factor and relative weight index, can be useful tools for identifying the most productive harvest areas and seasons and can therefore be employed to enhance the value and sustainability of the fishery. We recommend that consideration of fish condition cycles is incorporated into current fisheries management to achieve an optimal exploitation of fish stocks, concentrating on those seasons, areas, and size classes that maximize commercial benefit and minimize the impact of fishing mortality.

## Supporting Information

S1 Table Detailed biological data collection. Ref = Reference number of individuals. TL = Fish total length (cm). Wt = Fish total weight (g). LogTL = Natural logarithm of total length. LogWt = Natural logarithm of total weight. PredWt = Predicted weight based on LW equation from the best model. AntiWt = Anti log of the total weight. per75all = 75 percentile of fish weight in given length class. LogPer75 = Natural logarithm of per75all. LogWs = Natural logarithm of standard weight. Ws = individual standard weight. Wr = Relative weight. K = Condition factor. Sex = identified fish sex. MT = Monsoon Season. Month = Recorded of fish month caught. Year = Recorded of fish year caught. Period = Recorded of fish period caught. Zone = Recorded of fish zone caught. MA = Fish maturity category, Mature/immature. Depth = Recorded of water depth (m) where fish caught. Temp = Sea water temperatures (°C) where fish caught.
(CSV)
